# Population genomics of *Escherichia coli* in livestock-keeping households across a rapidly developing urban landscape

**DOI:** 10.1038/s41564-022-01079-y

**Published:** 2022-03-14

**Authors:** Dishon M. Muloi, Bryan A. Wee, Deirdre M. H. McClean, Melissa J. Ward, Louise Pankhurst, Hang Phan, Alasdair C. Ivens, Velma Kivali, Alice Kiyong’a, Christine Ndinda, Nduhiu Gitahi, Tom Ouko, James M. Hassell, Titus Imboma, James Akoko, Maurice K. Murungi, Samuel M. Njoroge, Patrick Muinde, Yukiko Nakamura, Lorren Alumasa, Erin Furmaga, Titus Kaitho, Elin M. Öhgren, Fredrick Amanya, Allan Ogendo, Daniel J. Wilson, Judy M. Bettridge, John Kiiru, Catherine Kyobutungi, Cecila Tacoli, Erastus K. Kang’ethe, Julio D. Davila, Samuel Kariuki, Timothy P. Robinson, Jonathan Rushton, Mark E. J. Woolhouse, Eric M. Fèvre

**Affiliations:** 1 Usher Institute, University of Edinburgh, Edinburgh, UK; 2 International Livestock Research Institute, Nairobi, Kenya; 3 Centre for Immunity, Infection and Evolution, University of Edinburgh, Edinburgh, UK; 4 Nuffield Department of Clinical Medicine, University of Oxford, John Radcliffe Hospital, Oxford, UK; 5 University of Nairobi, Nairobi, Kenya; 6 Kenya Medical Research Institute, Nairobi, Kenya; 7 Institute of Infection, Veterinary and Ecological Sciences, University of Liverpool, Neston, UK; 8 National Museums of Kenya, Nairobi, Kenya; 9 Research Center for Zoonosis Control, Hokkaido University, Sapporo, Japan; 10 Department of Epidemiology, Columbia University, New York, NY USA; 11 Veterinary Services Department, Kenya Wildlife Service, Nairobi, Kenya; 12 Uppsala University, Uppsala, Sweden; 13 Big Data Institute, Nuffield Department of Population Health, University of Oxford, Oxford, UK; 14 Natural Resources Institute, University of Greenwich, Chatham, UK; 15 African Population Health Research Centre, Nairobi, Kenya; 16 International Institute for Environment and Development, London, UK; 17 The Bartlett Development Planning Unit, Faculty of the Built Environment, University College London, London, UK; 18 Food and Agriculture Organization of the United Nations, Rome, Italy

**Keywords:** Infectious-disease epidemiology, Bacterial genomics

## Abstract

Quantitative evidence for the risk of zoonoses and the spread of antimicrobial resistance remains lacking. Here, as part of the UrbanZoo project, we sampled *Escherichia coli* from humans, livestock and peri-domestic wildlife in 99 households across Nairobi, Kenya, to investigate its distribution among host species in this rapidly developing urban landscape. We performed whole-genome sequencing of 1,338 *E. coli* isolates and found that the diversity and sharing patterns of *E. coli* were heavily structured by household and strongly shaped by host type. We also found evidence for inter-household and inter-host sharing and, importantly, between humans and animals, although this occurs much less frequently. Resistome similarity was differently distributed across host and household, consistent with being driven by shared exposure to antimicrobials. Our results indicate that a large, epidemiologically structured sampling framework combined with WGS is needed to uncover strain-sharing events among different host populations in complex environments and the major contributing pathways that could ultimately drive the emergence of zoonoses and the spread of antimicrobial resistance.

## Abstract (web summary)

Phylogeography and phylogenomic analyses of *E. coli* isolates collected from humans and domesticated and wild animals across 99 households in Nairobi reveal strong intra-household, and lower but detectable inter-household and inter-host, strain-sharing patterns.

## Main

The spread of bacterial pathogens and antimicrobial resistance (AMR) across human and animal populations presents a substantial and growing threat to global health and economic development. Identifying risk factors for emergence and spread is one of epidemiology’s most important challenges. Many recent pandemics and newly emergent infectious diseases have animal origins^1,2^ and are associated with rapidly urbanizing environments^3,4^. The dynamic interfaces among humans, domestic livestock and wild animals act as conduits by which humans can be exposed to zoonotic pathogens and AMR in an environment with inadequate sanitation infrastructure, limited access to appropriate and effective drugs and unregulated antimicrobial usage^5–8^.

The importance of livestock to the transmission of bacteria and AMR remains unclear^9^. The practice of keeping livestock, particularly in urban settings, has been described as a risk factor for the emergence and spread of zoonoses^10,11^. Antimicrobial agents used in human medicine are also used for growth promotion, disease prevention and disease treatment in livestock, enhancing selection pressures on bacterial pathogens for AMR emergence and spread.

Wild birds and mammals have also been documented to carry and exchange drug-resistant bacteria with livestock and humans^6,12,13^. The rapid expansion of urban environments into previously pristine or sparsely populated natural landscapes also increases the potential for greater contact among wildlife, humans and livestock, which can provide conduits for microbiome sharing^14^.

Fundamental to whole-genome sequencing (WGS) studies is the availability of systematically sampled bacterial isolates obtained from humans, livestock and wildlife across overlapping geographical regions and time frames, yet data are lacking^15^. In this study, we sampled the bacterium *Escherichia coli* from humans, livestock and peri-domestic wildlife in 99 households and their environs across 33 sublocations in Nairobi, Kenya, in an epidemiologically structured study. The rapid development of Nairobi’s urban landscape is similar to that of many other cities in the developing world, making it an ideal system in which to explore how people’s interactions and co-existence with animals influences pathogen transmission across species^16,17^.

This ‘99 households’ study was part of a broader study (‘Epidemiology, Ecology and Socio-Economics of Disease Emergence in Nairobi’, or ‘UrbanZoo’ for short) and focused on mechanisms for zoonotic pathogen emergence in urban environments. The broader study included mapping agriculture-sector value chains to understand the flow of animal source food products into the city of Nairobi^18–26^ as well as the aetiology of childhood diarrhoea in low-income settlements, studies quantifying antibiotic drug resistance carriage in multiple hosts^6,12^ and the roles of different hosts in disseminating clinically important resistance profiles^27,28^. It also included work to explicitly analyse the interplay among urbanization, food supply and pathogen risk^29^. The data presented here explore the phylogeography of bacterial isolates across an urban landscape.

As a common commensal and pathogen in vertebrates, as well as its ease of isolation and culture and its wealth of available genetic information, *E. coli* is an ideal exemplar bacterium to study the more general phenomenon of dispersal of pathogens across host populations. Here we report a genomic investigation of 1,338 *E. coli* isolates sourced from humans, livestock and wildlife across Nairobi to elucidate patterns of bacterial strain sharing as a proxy for transmission potential. We test the hypothesis that the distributions of bacterial strains and their genetic pools are limited to particular defined ecological niches (households and hosts) versus an alternative that they display a cosmopolitan distribution—in essence, recapitulating the famous tenet, “Everything is everywhere, but the environment selects”^30^. By considering both household and host factors, our study captures both neutral (dispersal limitation) and niche (environmental selection) processes in driving bacterial distribution^31^. Our study aims to identify risk factors to help inform surveillance strategies that target potential hotspots for strain sharing and AMR transmission among populations in an urban setting and, more broadly, to understand risks associated with transmission of multi-host pathogens in urban settings.

## Results

### *E. coli* in Nairobi are from both global and local lineages

**Fig. 1:**
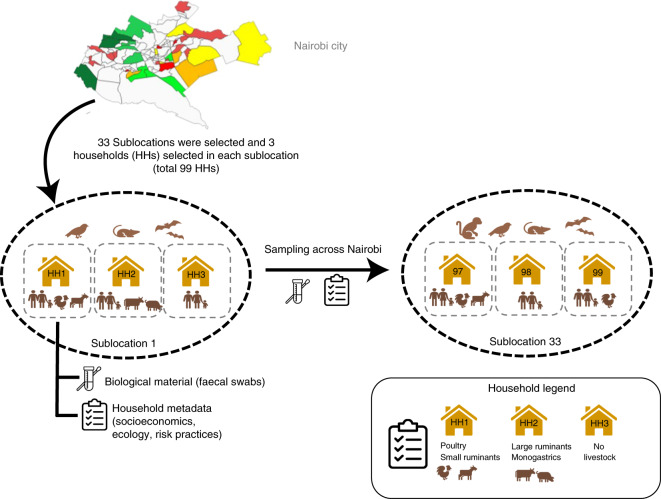
Flow diagram of the household selection procedure. Different colours given to the sublocations on the Nairobi city map represent different wealth categories (dark green, wealthy; dark red, poor). Source data

**Extended Data Fig. 1:**
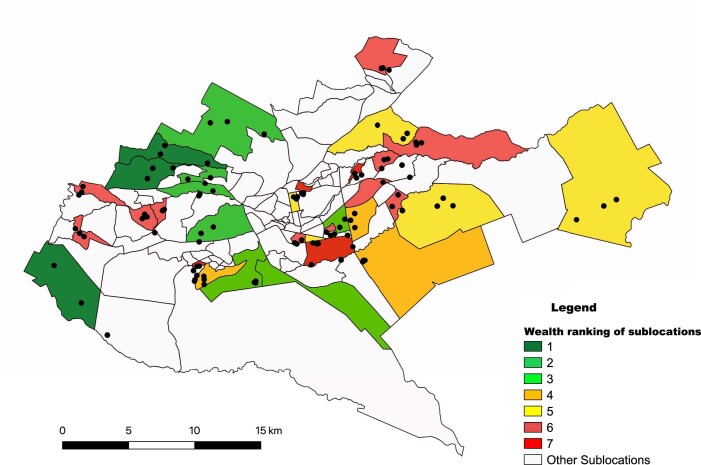
Geographical distribution of selected sublocations within the city of Nairobi chosen based on a socio-economic stratification, together with locations of each of 99 households selected within each stratum. Different colours given to the sublocations represent different wealth categories (Dark green – wealthy, dark red – poor). Source data

A total of 1,338 *E. coli* isolates were sequenced as part of this study (Supplementary Table 1). In total, 311 genomes were obtained from human isolates; 421 genomes were isolated from 63 wildlife species, primarily composed of wild birds (*n* = 245), rodents and bats (*n* = 130) isolates; and 606 genomes were obtained from 13 species of livestock that can be grouped into poultry (*n* = 324), goat and sheep (*n* = 109), cattle (*n* = 61), pig (*n* = 49) and rabbit (*n* = 38) isolates. The isolates were distributed across 99 households from 33 geographic sublocations, spanning the entire urban area of Nairobi, with each sublocation represented by 20–63 isolates (Fig. 1, Extended Data Fig. 1 and Supplementary Methods).

The genomes represent all major lineages of the *E. coli sensu stricto* phylogroup in addition to members of the cryptic clade I. The isolates belong to Clermont phylogroups B1 (45%), A (38%), B2 (6%), D (4%) and E (2%) and, to a lesser extent, clades C, F and G and clade I (<1%). Phylogroup A was strongly associated with humans (41% of human isolates) compared with the other host categories. In the livestock mammal, wild bird and wild mammal categories, genomes from phylogroup B1 were the most frequently isolated.

**Fig. 2:**
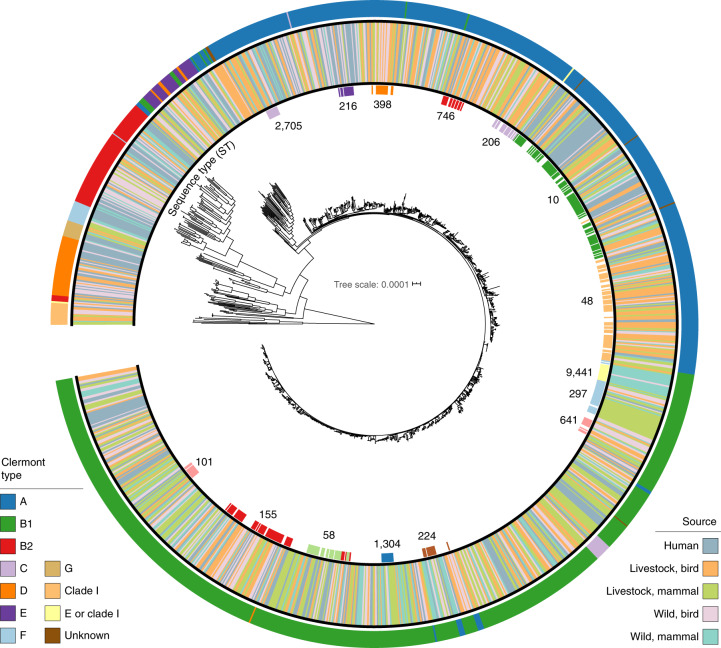
Core genome phylogeny of 1,338 *E. coli* isolates. Inner ring: STs (only STs with a minimum of ten isolates are shown); middle ring: source type of isolate; outer ring: Clermont phylotype classifications. The tree is rooted on the clade I group. Source data

**Extended Data Fig. 2:**
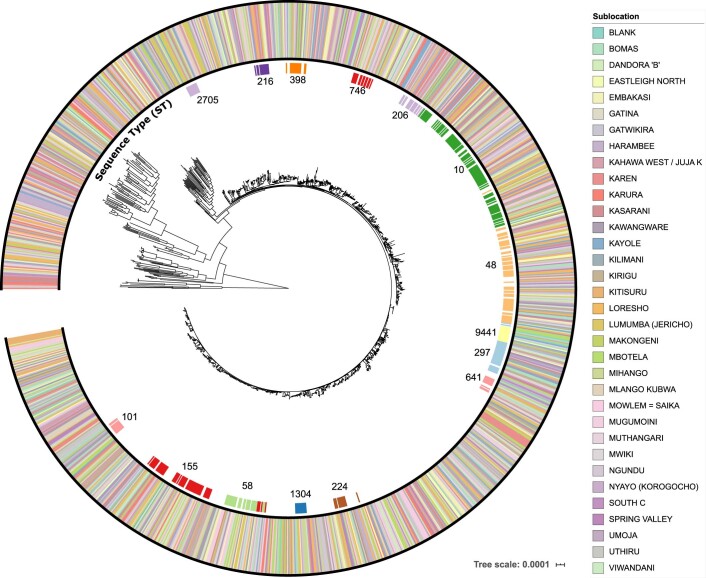
Maximum-Likelihood tree of a core genome alignment showing the distribution of isolates across 33 sublocations in Nairobi. The outermost ring is colored according to the sublocation of sample origin and innermost ring represents the commonly predicted sequence types (ST) in our isolate collection. Source data

A total of 537 sequence types (STs), based on the seven-gene Achtman scheme, were represented, with the three most common being ST10 (*n* = 93, 7%), ST48 (*n* = 64, 5%) and ST155 (*n* = 54, 4%) (Supplementary Table 2). One hundred and thirty-nine STs, representing 14% (184/1,338) of isolates, have been found only in African countries (Kenya, Madagascar, South Africa and Uganda), based on the genomes that were present in Enterobase at the time this study was carried out. One hundred and thirty-three of the Africa-specific STs in this collection, representing 13% (173/1,338) of the isolates, were unique to Kenya. Most of these novel and unique STs were isolated from livestock (52%, 96/184) and wildlife (34%, 63/184). A core-genome alignment comprising 80,722 nucleotide positions conserved across all 1,338 isolates was used to infer the overall phylogenetic relationship among isolates (Fig. 2). Additionally, we did not find extensive associations of isolates with either host species or sublocation (Fig. 2 and Extended Data Fig. 2).

### Clonal strain sharing of *E. coli*

**Fig. 3:**
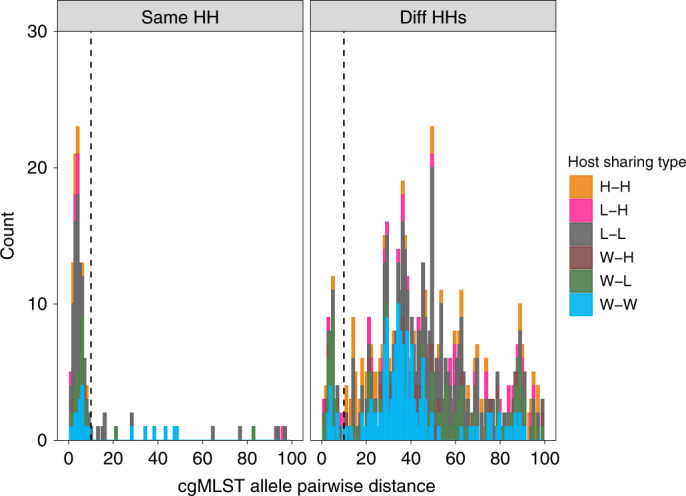
Frequency distribution of pairwise distances among isolates from the same household and from different households. cgMLST allele pairwise distances among isolates from the same household (HH; left) and from different (Diff) HHs (right). The sources of isolates in each pair are indicated by the colour. Only pairs that are closer than 100 cgMLST loci apart are shown. The vertical dashed black line indicates the sharing threshold (10 cgMLST alleles). H, human; L, livestock; W, wildlife. Source data

**Extended Data Fig. 3:**
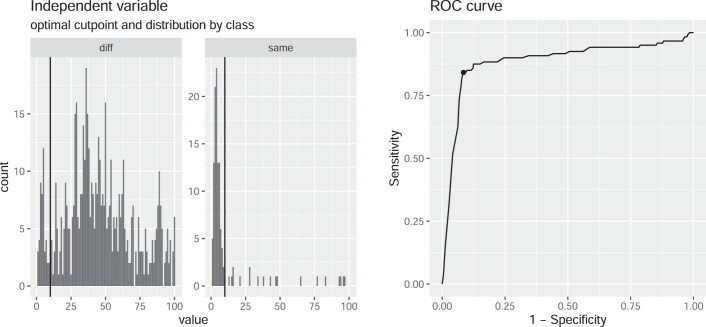
Output of cutpointR, used to identify the optimal threshold to maximise the differentiation between sharing pairs occurring within households (same) and between households (diff) at 10 cgMLST loci. The black vertical lines on the 2 panels on the left indicate the *optimal_cutpoin*t value of 10 as calculated by cutpointR. Source data

**Extended Data Fig. 4:**
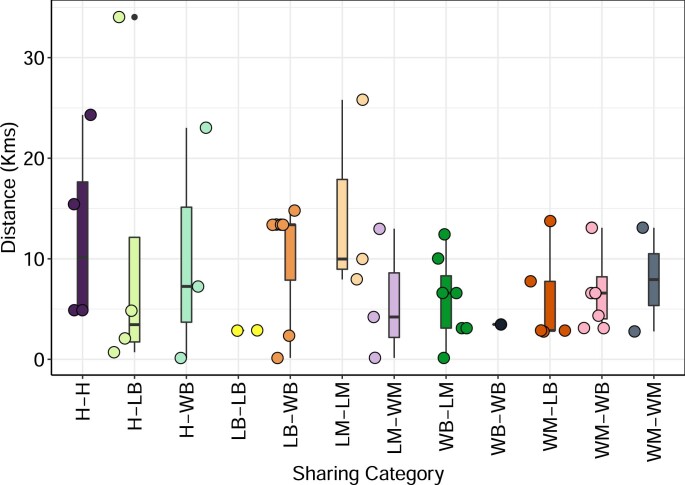
Plot of geographical distance of bacterial sharing in pairs involving different households (n = 49). Abbreviations: H- Human, LB – Livestock Bird, WB – Wild Birds, LM – Livestock mammals, LB – Livestock Birds. Boxplot centre lines show median value; upper and lower bounds show the 25th and 75th quantile, respectively; upper and lower whiskers show the largest and smallest values within 1.5 times the interquartile range above the 75th percentile and below the 25th percentile, respectively; and points show data points (jittered to improve visualisation). Source data

Transmission of bacteria, either directly or indirectly via a common source, can be inferred by the presence of very closely related genomes in two individuals, which we refer to as clonal strain sharing. To identify clonal strain sharing, we used core-genome, multi-locus sequence typing (cgMLST), which is a measure of genetic relatedness that is reproducible and scalable across larger and more diverse datasets^32^. We first plotted the frequency distribution of pairs of isolates differing by fewer than 100 cgMLST loci (Fig. 3). Here, we found a total of 150 pairs of isolates that differed by ten or fewer cgMLST alleles from other isolates in our collection. These pairs comprised 187 (14%) isolates, with some isolates involved in multiple pairs. Data on household and host type for these 150 pairs revealed that most occurred among hosts from the same household (*n* = 101, 67%) and 33% (*n* = 49) involved hosts from different households. Given the low genetic distances and epidemiological context, we refer to these pairs of ≤10 cgMLST loci as ‘sharing pairs’ to indicate evidence of recent strain sharing either by direct transmission or acquisition from a common source (Extended Data Fig. 3). We found no significant correlation between host type sharing and inter-household geographical distance (*χ*^2^ = 8.83, *P* = 0.64, Kruskal–Wallis) (Extended Data Fig. 4).

**Extended Data Fig. 5:**
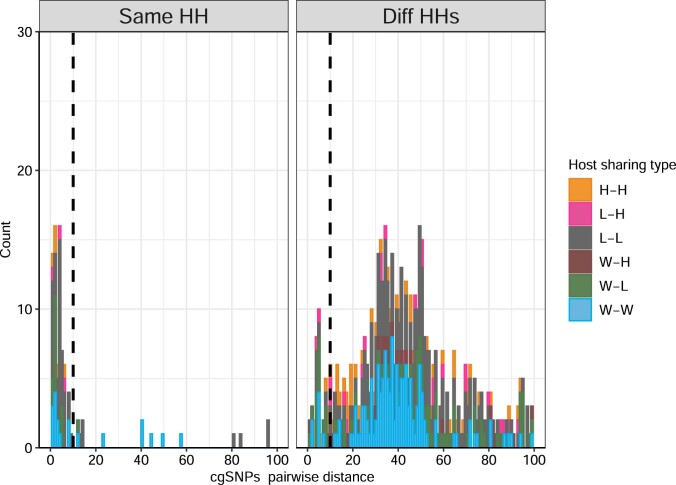
The distribution of closely related isolates (separated by 100 cgSNPS, n = 660) based on core genome SNPs (cgSNPs). An alignment of 399,673 aligned nucleotide positions were used. Each of these core SNP positions were conserved in at least 99.8% of isolates (1335/1338). The vertical dashed black line indicates the sharing threshold (10 cgSNPs). Source data

**Extended Data Fig. 6:**
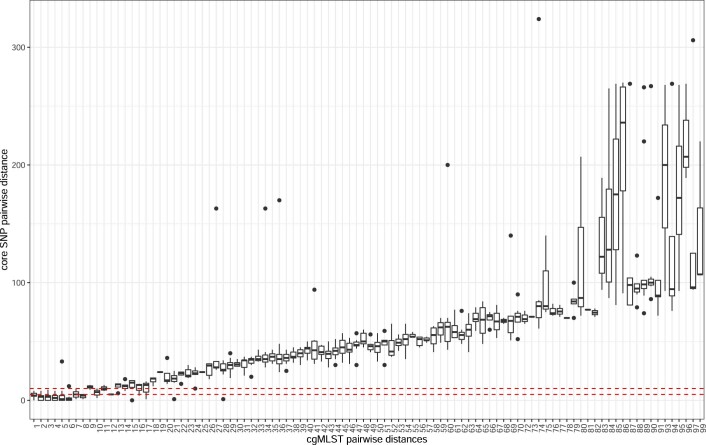
The correlation between pairwise genetic distances measured by core genome single nucleotide polymorphisms (cgSNPs) and core genome multi locus sequence typing (cgMLST) of pairs of isolates with ≤ 100 cgMLST and ≤ 1000 cgSNPs. 73% of pairs with ≤ 10 cgMLST loci apart (n = 109) were separated by ≤ 4 cgSNPs while 97% (n = 145) of these ≤10 cgMLST pairs were separated by ≤10 core SNPs. Only one pair had more than 13 cgSNPs. Two horizontal red dotted lines indicate 4 and 10 cgSNPs. Boxplot centre lines show median value; upper and lower bounds show the 25th and 75th quantile, respectively; upper and lower whiskers show the largest and smallest values within 1.5 times the interquartile range above the 75th percentile and below the 25th percentile, respectively; and points show show samples outside the whisker range. Source data

Pairwise core-genome, single-nucleotide polymorphisms (cgSNPs) of these sharing pairs were also investigated to validate the genetic distance as measured by cgMLST. The distribution of closely related pairs (<100 cgSNPs) also showed a similar pattern, with 159 pairs separated by fewer than ten cgSNPs (Extended Data Fig. 5). Both cgMLST and cgSNPs measures captured very closely related pairs of isolates, with 73% of the sharing pairs (*n* = 109) separated by four or fewer cgSNPs and 97% (*n* = 145) by a maximum of ten cgSNPs (Extended Data Fig. 6). Only one pair had more than 13 cgSNPs. WGS studies of *E. coli* outbreaks in humans have shown that epidemiologically linked isolates usually differ by up to four cgSNPs when isolated within 30 days of each other and, when separated by 5–10 core cgSNPs, this time frame increases to an average of 8 months^33^. Therefore, the genetic diversity of isolates within the same household agrees with examples of epidemiologically linked *E. coli* in other settings, and we estimate that length of evolutionary time separating two isolates from within the same household is within the range of several months to several years.

**Extended Data Fig. 7:**
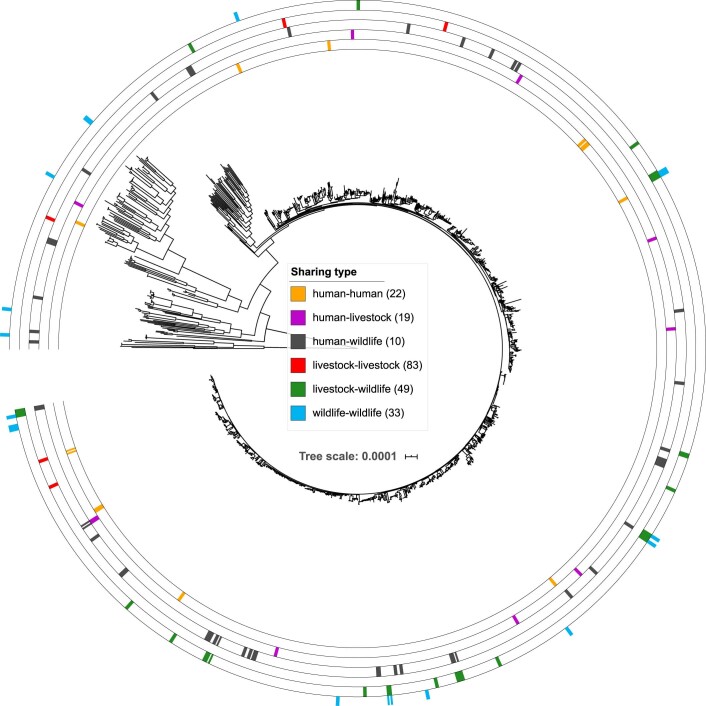
Core genome phylogenetic tree showing the distribution of isolates involved in the 6 different categories of sharing events. Ring colours indicate the sharing category (human-human, human-livestock, human-wildlife, livestock-livestock, livestock-wildlife, wildlife-wildlife) from the innermost to the outermost rings, respectively. Source data

Sixty-five percent (*n* = 97) of the pairs were between isolates from the same host category (57 (38%) within livestock, 26 (17%) within wildlife and 14 (9%) within humans), and the remaining 36% (*n* = 53) were found between host categories (38 (25%) between wildlife and livestock (W–L), ten (6%) between human and livestock (H–L) and five (3%) between human and wildlife (H–W)). Further details on the breakdown of these sharing pairs are provided in Supplementary Table 1. No correlation was evident between sharing pairs and particular *E. coli* lineages, as sharing pairs were distributed across the phylogeny for all six (H–H, L–H, L–L, W–H, W–L and W–W) categories of sharing (Extended Data Fig. 7). However, in seven cases, wildlife isolates that were implicated in sharing pairs were found in the same cluster as isolates involved in sharing pairs with other host categories (Extended Data Fig. 7).

### *E. coli* strain sharing between humans and livestock

We identified ten sharing pairs involving human and livestock isolates belonging to STs that were not host restricted and have been associated with a variety of sources and host species (Table 1).

**Table 1:** Details of humans involved in bacterial sharing with livestock (≤10 cgMLST loci)

Sharing pair	Livestock host	cgMLST distance	ST	Household	Livestock-keeping status	Human–livestock-handling status	Gender
1	Chicken	1	10	Different	Yes	Yes	Male
2	Goose	1	538	Same	Yes	Yes	Male
3	Chicken	3	23	Different	No	–	Male
4	Cattle	3	6,178	Same	Yes	Yes	Male
5	Duck	3	58	Same	Yes	Yes	Male
6	Rabbit	4	9,454	Same	Yes	Yes	Male
7	Turkey	4	9,454	Same	Yes	Yes	Male
8	Chicken	4	206	Same	Yes	–	Male
9	Turkey	8	1,237	Different	Yes	–	Male
10	Chicken	10	48	Different	No	None	Male

–, Information not collected

All sharing pairs involved human males (*P* = 0.003, Fisher’s exact test). Six of the ten sharing pairs involved humans and livestock in the same household, whereas four humans (not keeping livestock) shared bacteria with livestock from other households. The ten sharing events between humans and livestock did not always occur in a livestock-keeping household. Six of seven persons (we lacked data for three people) had direct contact with livestock through collecting eggs, slaughter, milking or handling, but one person had no history of livestock contact (Table 1).

### Sharing is shaped by host and households

**Fig. 4:**
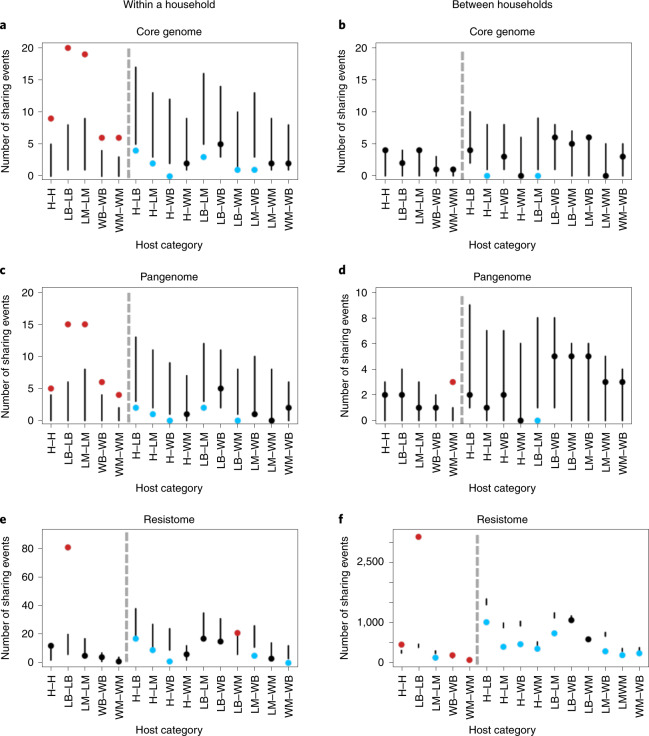
Number of sharing pairs for core genomes, pangenomes and resistomes within and between households. **a**,**c**,**e**, Number of within-household sharing pairs across 15 host category types for core genomes (**a**) (*n* = 121), pangenomes (**c**) (*n* = 94) and resistomes (**e**) (*n* = 9,502). **b**,**d**,**f**, Number of between-household sharing pairs across 15 host category types for core genomes (**b**) (*n* = 121), pangenomes (**d**) (*n* = 94) and resistomes (**f**) (*n* = 9,502). Panels show the 95% confidence intervals (vertical lines) of the calculated expected distribution using a resampling approach. Points depict the observed number of sharing pairs in each category coloured according to whether they fall above (red), below (blue) or within (black) the expected distribution. Hosts in the same category (for example, H–H) and different categories (for example, H–LB) are separated by grey dashed lines. Source type of isolate pairs is indicated on the *x* axis with human (H), livestock birds (LB), livestock mammals (LM), wildlife birds (WB) and wildlife mammals (WM). In each plot, within-category connections are on the left of the grey dotted line and between-category connections are on the right.

Household and host category strongly influenced the distribution of sharing of *E. coli* isolates in both the core genome and the pangenome in Nairobi (Fig. 4a–d). Within households, sharing of *E. coli* isolates was consistently higher than expected within the same host category (Fig. 4a,c). No strong pattern was observed among households where the observed shared *E. coli* isolates fell largely within the expected range (Fig. 4b,d). Resistome similarity was predominantly low among different hosts but high among poultry isolates, irrespective of household structure (Fig. 4e,f). Sharing among poultry (livestock birds (LB)) in the same household was particularly high across all three definitions of sharing and similarity—that is, the core, pangenome and resistome (LB–LB in Fig. 4).

**Extended Data Fig. 8:**
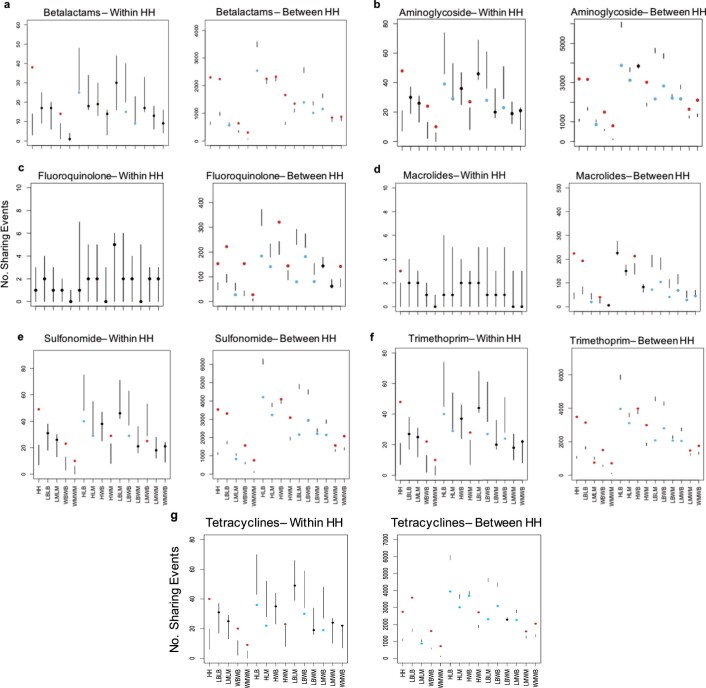
Number of sharing events across 15 host category types, within and between households for each of 7 identified antibiotic classes. (a) Beta-lactam (n = 21686), (b) Aminoglycosides (n = 36934), (c) Fluoroquinolone (n = 2088), (d) Macrolide (n = 1533), (e) Sulfonamide (n = 38125), (f) Trimethoprim (n = 36315), (g) Tetracyclines (n = 36816). Panels show the 95% confidence intervals (vertical lines) of the calculated expected distribution using a resampling approach, points depict the observed number of sharing events in each category coloured according to whether they fall above (red), below (blue) or within (black) the expected distribution. Source type of isolate pairs are indicated on the x-axis with either Human (H), Livestock birds (LB), Livestock mammals (LM), Wildlife birds (WB), Wildlife mammals (WM). In each plot, within-category connections are on the left of the grey dotted line and between-category connections are on the right. Source data

To further investigate resistome similarity between hosts, we performed the same analysis with sharing classed as two isolates sharing resistance genes that confer drug resistance to a given class of antibiotics. We compared eight classes of antibiotic whose resistance genes were found in the population (Extended Data Fig. 8) and found that, between households, poultry–poultry sharing continued to be much greater than the expected range (Extended Data Fig. 8). Resistome similarity among poultry does not, therefore, appear to be driven by resistance to a single or few antibiotic classes. H–H sharing between households was also higher than expected, suggesting similar antibiotic selection pressures on human isolates across the board.

## Discussion

Our population genomic analysis, explicitly embedded within an epidemiologically structured sampling framework, provides a comprehensive overview of the genomic landscape of *E. coli* in humans, livestock and peri-domestic wildlife in a rapidly developing city. Our findings have implications for understanding the baseline level of bacterial diversity in settings where there is a potential for interaction between humans and animals. Our results reveal strain sharing occurring within households and a lower but detectable level of connectivity among human and animal populations across the urban environment beyond the household.

Isolates from Africa make up less than 3% (*n* = 3,626) of the publicly available *E. coli* genome sequences in the public genome database, Enterobase. Our study provides a substantial contribution to the record of *E. coli* diversity in this part of the world with the identification of 133 unique and novel STs, in addition to a detailed footprint at a city-wide scale. Previous work on the population structure of *E. coli* isolated from human, livestock and wildlife in other both rural and urban settings showed varying degrees of overlap in the genotypes among these populations, driven by frequent contact and close proximity^13,14,34^. The wide range of genotyping methods used in these studies, each with varying levels of resolution, makes it difficult to make direct comparisons between studies. Earlier genotyping methods have lower resolution and are less robust^35^. Other studies measure similarity in microbiome community composition but are less reliable at resolving strain differences between samples^36^. Our approach combines high-resolution WGS with a structured sampling design, which captures more accurately the extent of strain sharing in this location.

In our study, we found that household stratification drives clonal strain sharing. Previous studies have shown an important role of the household as a driver for sharing similar microbiomes or bacteria in humans and companion animals^37–41^. Our findings show that strain sharing can involve humans, livestock and wildlife found in the same household or area.

The use of isolates collected within a time frame of 14 months in this study increased our ability of finding clonal isolates that overlap among hosts, households and sublocations. Previous work using whole genomes found either no overlap or isolates that were separated by more than ten cgSNPs, which does not provide strong evidence for a recent sharing event^42,43^. Although challenging in practice, we have demonstrated the importance of large-scale structured sampling to understand strain sharing at the population level.

Genotype similarity of the core and accessory genome within households is posited to be driven by direct and social contact among individual hosts^44,45^. Consistent with expectation, host type was also shown to be a strong driver in *E. coli* isolate sharing within households (Fig. 4). Members of the same host category, particularly in the same household, are more likely to have direct and/or indirect contact within shared environments, creating increased opportunity for bacterial sharing^14,36,37,44–46^.

Eight of the ten H–L strain-sharing events that we identified involved various poultry species. Inhalation and ingestion of faecal dust from poultry has previously been identified as a significant risk in the spread of bacteria from one host to another, both within the poultry populations and with humans working in close contact with them^47^. Furthermore, closely related ST131 strains have been previously found in both human and poultry *E. coli* populations, and genetic factors responsible for causing infections in chickens are also found in human pathogenic isolates^48–51^. Humans in direct contact with livestock were more prone to sharing *E. coli* isolates, probably through direct contact with livestock products and/or faecal matter. Although the sample size of such sharing events within our large overall sample is small, this result is consistent with previous work postulating direct contact as a risk for bacterial sharing^39,52^. The results also serve to highlight that detecting connections or common sources among pathogens in spatially distributed hosts in large, complex environments requires carefully structured sampling designs that account for the considerable heterogeneity in natural systems^53^. We note that the strong host-type signal for *E. coli* sharing within a household (Fig. 4a) does not hold true when examining pairs between households (Fig. 4b). This could be due to a higher diversity of *E. coli* in the wider population, leading to a lower probability of detecting closely related strains.

Our resistome similarity analysis also suggests disproportionately higher rates of resistome similarity among poultry, irrespective of the household, compared with the other host groups. As poultry isolates are phylogenetically diverse, the presence of a common selection pressure could explain this observation. Across Nairobi, poultry are routinely exposed to a set regimen of antimicrobial agents (for therapeutic or prophylactic purposes), and such recipes vary minimally geographically from one location to another^54^. Conversely, a wider range of combinations of antimicrobials is available for use in ruminants and monogastrics, including an array of injectable formulations, and these greatly vary from one farm to another. We also find resistome similarity to be higher than expected among human and wildlife isolates, both mammals and birds. The similar availability and usage patterns of antibiotics in the human population across the city could explain the similarity seen in humans, suggesting that resistome similarity occurs from prevailing selective pressures rather than spread from a common source. The presence of manure, rubbish and human waste—all contaminated with potentially similar kinds of AMR pathogens and antimicrobials—across the urban landscape of Nairobi provides a conduit for acquisition and/or selection of similar resistomes in wildlife, which act as a sink population for AMR^12^.

We observed a higher-than-expected level of accessory genome sharing among wild mammals (bats and rodents) and among households, apparently involving divergent lineages, as we did not see the same pattern at the core-genome level. Other types of wildlife (for example, wild birds) around the world have been shown to carry and transmit *E. coli* and should be considered a public health risk^55–57^. Our findings suggest that the role of rodents and bats should also be considered.

Our study design focuses on the breadth of sampling over depth, and, as a single isolate is sampled from each host, our approach does not account for intra-host diversity. Previous studies on the intra-host diversity of *E. coli* strains found them to be variable across host populations, and taking single isolates has the potential to underestimate the number of potential strain-sharing events^58^. However, our study using single isolates already reveals sharing events between human and animal hosts, and the scale of sharing can only be higher with incremental samples per host. Future studies should, therefore, consider both inter-host and intra-host diversity to expand on our findings.

## Conclusions

Employing an epidemiologically structured sampling framework and using highly discriminatory WGS, our study provides detailed insight into the strain diversity of *E. coli* across a fast-growing African city where livestock-keeping within households is commonplace. To our knowledge, this is one of the largest and most comprehensive surveys of the bacterial genomic landscape in an urban environment so far, and it serves as a model for epidemiologically structured, targeted sampling and WGS of human and animal-borne bacteria. We found evidence of recent clonal sharing between humans and livestock, and we show that the *E. coli* population structure in humans, livestock and wildlife in this environment is shaped by both household and host type. These findings indicate that household bacterial distribution is predominantly, although not exclusively, driven by dispersal limitation, whereas, within the household, the host niche is the strongest driver for bacterial sharing (and their genetic pools) distribution. We also found similarities in the resistome of the isolates that did not match the patterns of shared genomes and presumably reflects common antibiotic usage practices, particularly in poultry. This provides the strongest evidence in our study for direct selection acting on bacteria within a host (shared antibiotic environment). These findings provide empirical support for the hypothesis that ‘Everything is everywhere’ (frequent sharing of bacteria and AMR genes between households) but ‘environment selects’ (different households and hosts have different bacterial and resistome persistence). From a disease-control-policy perspective, our study highlights the need to undertake surveillance for emerging pathogens at the appropriate spatial scale (here, households) and to account for patterns of interconnectivity where epidemiological links might be created by livestock, wildlife or humans themselves. Further work, guided by the finding of where clonal sharing is most likely to be found, will be required to quantify spillover risk associated with the main routes of inter-host transmission.

## Methods

### Study site

A cross-sectional study targeting synanthropic wildlife and sympatric human and livestock populations in Nairobi, Kenya, was carried out from August 2015 to October 2016 as part of the UrbanZoo project. Faecal samples (*n* = 2,081) from 75 wildlife species (birds and mammals, *n* = 794), 13 livestock species (*n* = 677) and humans (*n* = 333) were collected from households across Nairobi that were participating in the UrbanZoo 99 households project. Our study design is described in detail in the Supplementary Methods. In brief, Nairobi was split into administrative units, and 33 were chosen based on a socioeconomic stratification, which was weighted by population, such that the larger proportion of low-income households was oversampled while ensuring representation of all other socioeconomic groups. Three households were randomly selected in each sublocation to obtain two livestock-keeping and one non-livestock-keeping household (a total of 99 households), with the aim of maximizing the spatial distribution and diversity of livestock-keeping practices captured within the sampling frame (Fig. 1 and Extended Data Fig. 1). Households in each sublocation had to meet strict inclusion criteria of keeping small mammals (rabbits) or poultry, large mammals (cattle, goats and sheep) or pigs or no livestock within the household perimeter. Wildlife samples were obtained by a range of taxon-specific trapping methods, which are described in the Supplementary Methods.

### Sample collection and microbiological testing

Questionnaires detailing household composition and socioeconomic data, as well as livestock ownership and management, were administered at each household using Open Data Kit Collect version 1.4.10 software^59^. Human, animal and wildlife faecal samples were collected and transported on ice to one of two laboratories (University of Nairobi or Kenya Medical Research Institute) within 5 h of collection. Samples were enriched in buffered peptone water for 24 h and thereafter plated onto eosin methylene blue agar (EMBA) and incubated for 24 h at 37 °C. Subsequently, five colonies were selected and subcultured on EMBA before being further subcultured on Müller–Hinton agar. A single colony was picked at random from the plate for each original sample (hereafter referred to as an ‘isolate’), and a 10-parameter biochemical test was used (triple sugar iron agar = 4, Simmon’s citrate agar = 1, and motility-indole-lysine media = 3, urease production from urea media = 1, oxidase from tetra-methyl-*p*-phenylenediamine dihydrochloride = 1) for identification of *E coli*.

### WGS

DNA was extracted from bacterial isolates using commercial kits (Purelink Genomic DNA Mini Kit, Invitrogen, Life Technologies) at the International Livestock Research Institute in Nairobi, Kenya, and transported under licence to the Wellcome Trust Centre for Human Genetics. WGS was carried out at the Wellcome Trust Centre for Human Genetics on the Illumina HiSeq 2500 platform.

### Sequence analysis

Sequenced reads were filtered for quality and trimmed for adaptors with BBDuk (version 38.46), using k = 19, mink = 11, hdist = 1, ktrim = r, minoverlap =12, qtrim = rl and trimq = 15. The following sequencing quality thresholds were used based on Quast: (1) at least 3 Mb aligned to EC958; (2) a maximum assembly length of 6.5 Mb; (3) GC content of between 50% and 51%; and (4) assembly N50 of >30 kb or a maximum of 100 cgMLST missing loci. In total, 1,642 genomes were sequenced that passed this quality threshold.

Genomes were assembled using Spades version 3.13.0 with the ‘–careful’ option. Clermont phylotype of the isolates was determined using the ClermonTyping tool version 1.4.1^60^, and the multi-locus sequence type was determined and assigned by Enterobase^61^.

The pangenome was estimated using Roary version 3.12.0 with the following options: -s -i 95 -g 100000. Acquired antibiotic resistance genes were identified from the assemblies using starAMR (version 0.4.0) (https://github.com/phac-nml/staramr), with a cutoff of 95% sequence identity and a minimum of 60% alignment to the query sequence, against the ResFinder database downloaded on 25 September 2019^62^. Antibiotic class of each resistant gene was assigned using the ResFinder classification.

### Phylogenetic analyses

A core genome alignment was generated using Snippy version 4.6.0 (with default settings) using EC958 as a reference genome (GCA_000285655.3). A phylogenetic analysis of the core genome alignment was performed using IQTREE (version 1.6.12) -m TVM + G4 -bb 1000 -safe. The tree and metadata were visualized in iToL version 4.3 (itol.embl.de). Owing to the large number of isolates and the high level of diversity, we did not mask recombinant regions of the genome.

Ad hoc cgMLST was performed on genome assemblies using chewBBACA (v. 2.0.11) with the 2,513 gene cgMLST profile from Enterobase (downloaded October 2018).

### Identification of putative bacterial sharing

A genetic distance matrix was calculated from all pairwise-allelic-profile comparisons using the library ‘ape’ in R (ref. ^63^). The cgMLST cutoff of 11 alleles to define putative *E. coli* (defined here as a sharing pair) transmission clusters was based on the observed bimodal distributions of inter-household and intra-household allele differences (Extended Data Fig. 3). The R package ‘cutpointR’ was used to validate this cutoff as the optimal value to differentiate pairs that occur within and between households^64^. Pairwise cgSNPs were also calculated using the full consensus genome alignment generated by Snippy version 4.6.0 (snippy-core), followed by custom filtering positions that were fully called and unambiguous with an A, G, C or T that were conserved in at least 99.8% (1,335 of 1,338) of isolates (length = 399,673 nucleotides). Pairwise distances were calculated using Disty McMatrixface version 0.1.0 (https://github.com/c2-d2/disty) with -n 0.002.

### Epidemiological analysis of sharing

We established epidemiological links between every possible pair of *E. coli* isolates through a systematic comparison. Household-level sharing was categorized as within-household if a sharing pair involved isolates/hosts from the same household and between-household if a sharing pair involved isolates from a different household. Wildlife isolates that could not be attributed to a specific household were omitted from the sharing analysis (Supplementary Table 2).

We condensed our host types into five broad categories (Supplementary Tables 1 and 2): (1) humans; (2) livestock birds, poultry dominated by chickens; (3) livestock mammals, consisting of ruminants and monogastric livestock, (4) wild birds, predominantly seed-eating birds such as house sparrows; and (5) wild mammals, predominantly rodents, along with bats. Primates were omitted from the sharing analysis as they were associated with only two households, along with some samples derived from populations of bats and wild birds, which could be attributed to sublocation but not household.

Although the sharing threshold for the core genome was ≤10 cgMLST distance, sharing for the pangenome and resistome similarity was based on a Jaccard similarity index (JI) (between 0 and 1, where 1 is identical), where a cutoff threshold was defined, similar to the core genome. For the pangenome/accessory genome, this was determined to be JI ≤ 0.98 (Fig. 3c,d). Resistome sharing was defined as JI = 1 (Fig. 3e,f), with each isolate having a minimum of two AMR genes. In practice, this means that two isolates must share an identical set of AMR genes of length ≥2.

To calculate the number of observed sharing events, we identified clusters of isolates that were within the sharing threshold. So as to count an isolate as ‘shared’ only once for clusters >2, we applied a Hamiltonian path method^65^ such that the number of pairs/connections is counted as *m* − 1, where *m* is the number of isolates that form a cluster (Supplementary Fig. 9).

Having defined the number of observed sharing events among each of our host categories within and between households, we then wanted to know whether these observed events fell above or below what might be expected given the differential sampling effort across host categories. To do this, we first calculated the total number of possible pairs, assuming equal chance of sharing. Within households, this was calculated using the formula *n*(*n*−1)/2, where *n* is the number of samples of a given host type within a household. Between-household sharing was calculated as (*n*_1_) × (*n*_2_), where *n*_1_ is the number of samples of a given host in household 1, and *n*_2_ is the number of samples of a given host in household 2. These values were then calculated as a proportion of the total number of all possible pairwise combinations. We next performed a simulation to see how the observed sharing events were distributed, given the proportion of each pairwise host combination calculated in the previous step.

To do this, we resampled (using the rmultinom function) the total number of observed values for each type of sharing (resampling with replacement 1,000 times) from the calculated proportions. These resampled values were then used to generate the expected range of sharing events (± 95% confidence intervals) for each pairwise combination of host category. From this, we were able to assess whether our observed sharing events fell above, below or within the range that we might expect given the sampling effort. This pattern of sharing events among hosts and households enabled us to highlight cases where we observed sharing among hosts that lay outside from the predicted range. The same approach was applied to all aspects of genome sharing (Fig. 3a–f).

### Ethical approval

The collection of data adhered to the legal requirements of the Government of Kenya. The International Livestock Research Institute (ILRI) Institutional Research Ethics Committee is registered and accredited by the National Commission for Science, Technology and Innovation in Kenya and is approved by the Federalwide Assurance for the Protection of Human Subjects in the United States. Ethical approval for human sampling and data collection was obtained from the ILRI Institutional Research Ethics Committee (ILRI-IREC2015/09). Livestock samples were obtained under the approval of the ILRI Institutional Animal Care and Use Committee (reference ILRI-IACUC2015/18), and permits were obtained from the Directorate of Veterinary Services. Wildlife were trapped under approval of an ILRI Institutional Animal Care and Use Protocol (IACUC2015/12), and permits were obtained from the National Museums of Kenya and Kenya Wildlife Service. Written informed consent was obtained from all adult participants and from the parents of underage participants.

### Reporting Summary

Further information on research design is available in the Nature Research Reporting Summary linked to this article.

## Supplementary information

Supplementary Information Supplementary Methods.

Reporting Summary.

Supplementary Tables Supplementary Table 1. Complete list of genomes and accession numbers generated in this study. Supplementary Table 2. Host categories and their constituents and sample numbers. Supplementary Table 3. Additional information on isolates involved in human–livestock sharing events.

## Data Availability

Whole-genome sequences used in this study are available under the BioProjects with accession numbers PRJEB32607 and PRJEB41827. The reference genome used for mapping is *E. coli* strain EC958 (GCA_000285655.3). The ResFinder AMR gene database used was downloaded on 25 September 2019 from https://bitbucket.org/genomicepidemiology/resfinder_db. Source data are provided with this paper.

## Source data

Source Data Fig. 1 Metadata (isolate source, Clermont phylotype and MLST) and Newick tree for Fig. 1.
Source Data Fig. 2 List of sharing pairs, cgMLST pairwise distance, host type and household sharing category.
Source Data Fig. 3 List of observed and expected sharing events for each household and host type for core genome, pangenome and resistome.
Source Data Extended Data Fig. 1 List of households and corresponding sublocation, wealth group and coordinates.
Source Data Extended Data Fig. 2 Metadata (household ID and MLST) and Newick tree for Extended Data Fig. 2.
Source Data Extended Data Fig. 3 Summary output of threshold analysis from cutpointR.
Source Data Extended Data Fig. 4 Household pair, host sharing category and geographical distance between household (KMs).
Source Data Extended Data Fig. 5 List of sharing pairs, cgSNP pairwise distance, host type and household sharing category.
Source Data Extended Data Fig. 6 List of pairwise cgMLST (<100) and cgSNP (<500) distances.
Source Data Extended Data Fig. 7 Lists of isolates involved in each type of sharing (H–H, H–L, L–L, H–W, L–W and W–W). H, human; L, livestock; W, wildlife.
Source Data Extended Data Fig. 8 List of observed and expected sharing events for each household and host type across seven antibiotic classes.

## Code availability

The scripts used to perform this analysis can be found at https://git.ecdf.ed.ac.uk/epigroup/urbanzoo.

## References

[1] Karesh WB (2012). Ecology of zoonoses: natural and unnatural histories. Lancet.

[2] Wolfe ND, Dunavan CP, Diamond J (2007). Origins of major human infectious diseases. Nature.

[3] Allen T (2017). Global hotspots and correlates of emerging zoonotic diseases. Nat. Commun..

[4] Zhang, F. et al. Global discovery of human-infective RNA viruses: a modelling analysis. *PLoS Pathog*. 10.1371/journal.ppat.1009079 (2020). 10.1371/journal.ppat.1009079 PMC7728385 33253277

[5] Gottdenker NL, Streicker DG, Faust CL, Carroll CR (2014). Anthropogenic land use change and infectious diseases: a review of the evidence. EcoHealth.

[6] Muloi D (2019). Epidemiology of antimicrobial-resistant *Escherichia coli* carriage in sympatric humans and livestock in a rapidly urbanizing city. Int. J. Antimicrob. Agents.

[7] Santiago-Alarcon, D. & MacGregor-Fors, I. Cities and pandemics: urban areas are ground zero for the transmission of emerging human infectious diseases. *J. Urban Ecol*. 10.1093/jue/juaa012 (2020).

[8] Woolhouse M, Ward M, van Bunnik B, Farrar J (2015). Antimicrobial resistance in humans, livestock and the wider environment. Phil. Trans. R. Soc. B.

[9] Hanage, W. P. Two health or not two health? That is the question. *mBio*10.1128/mBio.00550-19 (2019). 10.1128/mBio.00550-19 PMC6456756 30967467

[10] Hassell JM, Begon M, Ward MJ, Fèvre EM (2017). Urbanization and disease emergence: dynamics at the wildlife–livestock–human interface. Trends Ecol. Evol..

[11] Neiderus, C.-J. How urbanization affects the epidemiology of emerging infectious diseases. I*nfect. Ecol. Epidemiol*. 10.3402/iee.v5.27060 (2015). 10.3402/iee.v5.27060 PMC4481042 26112265

[12] Hassell JM (2019). Clinically relevant antimicrobial resistance at the wildlife–livestock–human interface in Nairobi: an epidemiological study. Lancet Planet. Health.

[13] Vittecoq M (2016). Antimicrobial resistance in wildlife. J. Appl. Ecol..

[14] Rwego IB, Isabirye‐Basuta G, Gillespie TR, Goldberg TL (2008). Gastrointestinal bacterial transmission among humans, mountain gorillas, and livestock in Bwindi Impenetrable National Park, Uganda. Conserv. Biol..

[15] Wee, B. A., Muloi, D. M. & van Bunnik, B. A. D. Quantifying the transmission of antimicrobial resistance at the human and livestock interface with genomics. *Clin. Microbiol. Infect*. 10.1016/j.cmi.2020.09.019 (2020). 10.1016/j.cmi.2020.09.019 PMC7721588 32979568

[16] Jaureguy F (2008). Phylogenetic and genomic diversity of human bacteremic *Escherichia coli* strains. BMC Genomics.

[17] Tenaillon O, Skurnik D, Picard B, Denamur E (2010). The population genetics of commensal *Escherichia coli*. Nat. Rev. Microbiol..

[18] Murungi, M. K. et al. The Nairobi pork value chain: mapping and assessment of governance, challenges, and food safety issues. *Front. Vet. Sci*. 10.3389/fvets.2021.581376 (2021). 10.3389/fvets.2021.581376 PMC7902891 33644142

[19] Kiambi S (2020). Investigation of the governance structure of the Nairobi dairy value chain and its influence on food safety. Prev. Vet. Med..

[20] Muloi D (2018). Value chain analysis and sanitary risks of the camel milk system supplying Nairobi city, Kenya. Prev. Vet. Med..

[21] Kiambi S (2018). Mapping Nairobi’s dairy food system: An essential analysis for policy, industry and research. Agric. Syst..

[22] Carron M (2018). Campylobacter, a zoonotic pathogen of global importance: prevalence and risk factors in the fast-evolving chicken meat system of Nairobi, Kenya. PLoS Negl. Trop. Dis..

[23] Onono JO (2018). Identification of production challenges and benefits using value chain mapping of egg food systems in Nairobi, Kenya. Agric. Syst..

[24] Alarcon, P. et al. Urban livestock keeping in the city of Nairobi: diversity of production systems, supply chains, and their disease management and risks. *Front. Vet. Sci*. 10.3389/fvets.2017.00171 (2017). 10.3389/fvets.2017.00171 PMC5669286 29164137

[25] Carron M (2017). The broiler meat system in Nairobi, Kenya: using a value chain framework to understand animal and product flows, governance and sanitary risks. Prev. Vet. Med..

[26] Alarcon P (2017). Mapping of beef, sheep and goat food systems in Nairobi—a framework for policy making and the identification of structural vulnerabilities and deficiencies. Agric. Syst..

[27] Hassell JM (2019). Deterministic processes structure bacterial genetic communities across an urban landscape. Nat. Commun..

[28] Hassell JM (2021). Socio-ecological drivers of vertebrate biodiversity and human-animal interfaces across an urban landscape. Glob. Change Biol..

[29] Ahmed S (2019). Participatory mapping and food-centred justice in informal settlements in Nairobi. Kenya Geo Geogr. Environ..

[30] Becking, L. B. *Geobiologie of inleiding tot de milieukunde* (WP Van Stockum & Zoon, 1934).

[31] Hubbell, S. P. *The Unified Neutral Theory of Biodiversity and Biogeography (MPB-32)* (Princeton Univ. Press, 2011). 10.1016/j.tree.2011.03.024 21561679

[32] Schürch AC, Arredondo-Alonso S, Willems RJL, Goering RV (2018). Whole genome sequencing options for bacterial strain typing and epidemiologic analysis based on single nucleotide polymorphism versus gene-by-gene-based approaches. Clin. Microbiol. Infect..

[33] Dallman TJ (2015). Whole-genome sequencing for national surveillance of Shiga toxin–producing *Escherichia coli* O157. Clin. Infect. Dis..

[34] Goldberg TL (2007). Patterns of gastrointestinal bacterial exchange between chimpanzees and humans involved in research and tourism in western Uganda. Biol. Conserv..

[35] Salipante SJ (2015). Application of whole-genome sequencing for bacterial strain typing in molecular epidemiology. J. Clin. Microbiol..

[36] Brito IL (2019). Transmission of human-associated microbiota along family and social networks. Nat. Microbiol..

[37] Lax S (2014). Longitudinal analysis of microbial interaction between humans and the indoor environment. Science.

[38] Mohamed M (2020). Large fecal reservoir of *Escherichia coli* sequence type 131-H30 subclone strains that are shared within households and resemble clinical ST131-H30 isolates. J. Infect. Dis..

[39] Mosites E (2017). Microbiome sharing between children, livestock and household surfaces in western Kenya. PLoS One.

[40] Naziri Z, Derakhshandeh A, Firouzi R, Motamedifar M, Tabrizi AShojaee (2016). DNA fingerprinting approaches to trace *Escherichia coli* sharing between dogs and owners. J. Appl. Microbiol..

[41] Domman D (2018). Defining endemic cholera at three levels of spatiotemporal resolution within Bangladesh. Nat. Genet..

[42] Falgenhauer L (2016). Circulation of clonal populations of fluoroquinolone-resistant CTX-M-15-producing *Escherichia coli* ST410 in humans and animals in Germany. Int. J. Antimicrob. Agents.

[43] Ludden, C. et al. One health genomic surveillance of *Escherichia coli* demonstrates distinct lineages and mobile genetic elements in isolates from humans versus livestock. *mBio*10.1128/mBio.02693-18 (2019). 10.1128/mBio.02693-18 PMC6343043 30670621

[44] Moeller AH (2016). Social behavior shapes the chimpanzee pan-microbiome. Sci. Adv..

[45] Tung J (2015). Social networks predict gut microbiome composition in wild baboons. eLife.

[46] Springer A, Mellmann A, Fichtel C, Kappeler PM (2016). Social structure and *Escherichia coli* sharing in a group-living wild primate, Verreaux’s sifaka. BMC Ecol..

[47] Tobin MR, Goldshear JL, Price LB, Graham JP, Leibler JH (2015). A framework to reduce infectious disease risk from urban poultry in the United States. Public Health Rep..

[48] Liu, C. M. et al. *Escherichia coli* ST131-H22 as a foodborne uropathogen. *mBio*10.1128/mBio.00470-18 (2018). 10.1128/mBio.00470-18 PMC6113624 30154256

[49] Roer L (2019). ST131 fimH22 *Escherichia coli* isolate with a blaCMY-2/IncI1/ST12 plasmid obtained from a patient with bloodstream infection: highly similar to *E. coli* isolates of broiler origin. J. Antimicrob. Chemother..

[50] Mageiros L (2021). Genome evolution and the emergence of pathogenicity in avian *Escherichia coli*. Nat. Commun..

[51] McNally, A. et al. Combined analysis of variation in core, accessory and regulatory genome regions provides a super-resolution view into the evolution of bacterial populations. *PLoS Genet*. 10.1371/journal.pgen.1006280 (2016). 10.1371/journal.pgen.1006280 PMC5019451 27618184

[52] Song SJ (2013). Cohabiting family members share microbiota with one another and with their dogs. eLife.

[53] VanderWaal KL, Ezenwa VO (2016). Heterogeneity in pathogen transmission: mechanisms and methodology. Funct. Ecol..

[54] Muloi D (2019). A cross-sectional survey of practices and knowledge among antibiotic retailers in Nairobi, Kenya. J. Glob. Health.

[55] Bonnedahl J (2009). Dissemination of *Escherichia coli* with CTX-M Type ESBL between humans and yellow-legged gulls in the south of France. PLoS ONE.

[56] Kauffman MD, LeJeune J (2011). European starlings (*Sturnus vulgaris*) challenged with *Escherichia coli* O157 can carry and transmit the human pathogen to cattle. Lett. Appl. Microbiol..

[57] Mukerji S (2019). Resistance to critically important antimicrobials in Australian silver gulls (*Chroicocephalus novaehollandiae*) and evidence of anthropogenic origins. J. Antimicrob. Chemother..

[58] Stoesser N (2015). Extensive within-host diversity in fecally carried extended-spectrum-beta-lactamase-producing *Escherichia coli* isolates: implications for transmission analyses. J. Clin. Microbiol..

[59] Hartung, C. et al. Open data kit: tools to build information services for developing regions. In *Proc. 4th ACM/IEEE International Conference on Information and Communication Technologies and Development*10.1145/2369220.2369236 (2010).

[60] Beghain J, Bridier-Nahmias A, Le Nagard H, Denamur E, Clermont O (2018). ClermonTyping: an easy-to-use and accurate in silico method for *Escherichia* genus strain phylotyping. Microb. Genomics.

[61] Zhou, Z. et al. The EnteroBase user’s guide, with case studies on Salmonella transmissions, Yersinia pestis phylogeny, and Escherichia core genomic diversity. *Genome Res*. 10.1101/gr.251678.119 (2020). 10.1101/gr.251678.119 PMC6961584 31809257

[62] Zankari E (2012). Identification of acquired antimicrobial resistance genes. J. Antimicrob. Chemother..

[63] Paradis E, Claude J, Strimmer K (2004). APE: analyses of phylogenetics and evolution in R language. Bioinformatics.

[64] Thiele C, Hirschfeld G (2021). Cutpointr: improved estimation and validation of optimal cutpoints in R. J. Stat. Softw..

[65] Alhalabi W, Kitanneh O, Alharbi A, Balfakih Z, Sarirete A (2016). Efficient solution for finding Hamilton cycles in undirected graphs. SpringerPlus.

