# Supplementary material for: Population genomics of Escherichia coli in livestock-keeping households across a rapidly developing urban landscape
Source: Nat Microbiol. 2022 Mar 14;7(4):581–9. doi: 10.1038/s41564-022-01079-y (PMC8975746; doi:10.1038/s41564-022-01079-y)
Supplement: Supplementary file 1 — Supplementary Methods. [file 41564_2022_1079_MOESM1_ESM.pdf]

nature portfolio

https://doi.org/10.1038/s41564-022-01079-y

# Supplementary information

# Population genomics of *Escherichia coli* in livestock-keeping households across a rapidly developing urban landscape

In the format provided by the authors and unedited

**Supplementary Methods**

# Population genomics of *Escherichia coli* in livestock-keeping households across a rapidly developing urban landscape.

Dishon M. Muloi[1,2,3+], Bryan A. Wee[1+], Deirdre M. H. McClean[1], Melissa J. Ward[3,4], Louise Pankhurst[4], Hang Phan[4], Alasdair Ivens[3], Velma Kivali[2], Alice Kiyonga[2], Christine Ndinda[2], Nduhiu Gitahi[5], Tom Ouko[6], James M. Hassell[2,7], Titus Imboma[8], James Akoko[2], Maurice K. Murungi, Samuel M. Njoroge[6], Patrick Muinde[2], Yukiko Nakamura[9], Lorren Alumasa[2], Erin Furmaga[10], Titus Kaitho[11], Elin M. Öhgren[12], Fredrick Amanya[2], Allan Ogendo[2], Daniel J. Wilson[13], Judy M. Bettridge [14], John Kiiru[6], Catherine Kyobutungi[15], Cecila Tacoli[16], Erastus K. Kang'ethe[5], Julio D. Davila[17], Samuel Kariuki[6], Timothy P. Robinson[18], Jonathan Rushton[7], Mark E. J. Woolhouse[1*%], Eric M. Fèvre[2,7*%]

[+] These authors contributed equally

[%] These authors contributed equally* Corresponding authors

## Study design

The UrbanZoo project, funded by the Medical Research Council, aimed to understand the mechanisms of disease introduction and emergence in human populations in a major developing city, Nairobi, Kenya, in the context of livestock keeping. A significant component of the UrbanZoo project was the '99 household project' which focused on sampling of households across socio-economic strata of Nairobi to investigate the role of informal livestock keeping practices as a route of zoonotic disease emergence in humans. As such, a cross-sectional study targeting sympatric human and livestock populations in Nairobi, was carried out from August 2015 to October 2016. Disaggregated socio-economic geospatial data at a much finer scale produced by Institut Français de Recherche en Afrique (IFRA) were used to identify 17 classes of residential neighbourhoods in Nairobi based on different land-use patterns (e.g. residential, industrial, institutional) and physical landscape attributes (such as tree cover, plot size, amount of gated space, roofing type, presence of agriculture). Each of the seventeen classes of neighbourhood were ranked by average income and merged into seven wealth groups (Table 1).

Table 1. Distribution of the seven wealth groups used by the Urban Zoo Project, and the number of sub-locations with a dominant wealth group identified and selected across the city.

<table>
<tr><th></th><th colspan="4">Characteristics of physical neighbourhood classes identified by IFRA study (adapted from Ledant, 2011[#])</th><th colspan="3">Urban Zoo Project re-classification</th></tr>
<tr><th>Code</th><th>Tree cover</th><th>Defining characteristics</th><th>Neighbourhood description</th><th>Av. monthly income per capita ($)</th><th>Wealth group</th><th>Possible sub-locations</th><th>Targeted sub-locations</th></tr>
<tr><td>A</td><td rowspan="2">> 13.5%</td><td rowspan="2">Detached housing with intense tree cover</td><td>Detached housing on very large plots (>3000 m²)</td><td>399</td><td>1</td><td>8</td><td>3</td></tr>
<tr><td>B</td><td>Detached housing on large plots (400 - 3000 m²)</td><td>225</td><td>2</td><td rowspan="3">8</td><td rowspan="3">4</td></tr>
<tr><td>C</td><td>> 13.5%</td><td>Attached and semi-detached housing</td><td>Attached housing on medium plots (<400 m²) with important tree cover</td><td>221</td><td>2</td></tr>
<tr><td>D</td><td>3-13.5%</td><td>Apartment building</td><td>Apartment buildings with gated space</td><td>221</td><td>2</td></tr>
<tr><td>E</td><td rowspan="2">3-13.5%</td><td rowspan="2">Attached and semi-detached housing</td><td>Higher standing row houses (plot size > 190 m²)</td><td>134</td><td>3</td><td>5</td><td>3</td></tr>
<tr><td>F</td><td>Lower standing row houses (plot size < 190 m²)</td><td>62</td><td>4</td><td rowspan="2">3</td><td rowspan="2">3</td></tr>
<tr><td>G</td><td rowspan="2"><3%</td><td rowspan="2">Roof cover >50% tiles</td><td>Lower standing apartment buildings</td><td>62</td><td>4</td></tr>
<tr><td>H</td><td>New areas of dense single housing development</td><td>39</td><td>5</td><td rowspan="4">9</td><td rowspan="4">5</td></tr>
<tr><td>J</td><td><3%</td><td>Roof cover > 40% concrete</td><td>High density multi-storey buildings</td><td>39</td><td>5</td></tr>
<tr><td>K</td><td>3-13.5%</td><td>Apartment building</td><td>Apartment buildings with open access</td><td>39</td><td>5</td></tr>
<tr><td>L</td><td rowspan="2">3-13.5%</td><td rowspan="2">Peripheral areas</td><td>Peripheral areas (mainly residential)</td><td>39</td><td>5</td></tr>
<tr><td>M</td><td>Peripheral areas with rural component (presence of agriculture)</td><td>22</td><td>6</td><td rowspan="5">24</td><td rowspan="5">11</td></tr>
<tr><td>N</td><td rowspan="2">3-13.5%</td><td rowspan="2">Collective housing</td><td>Community housing with gated space</td><td>22</td><td>6</td></tr>
<tr><td>P</td><td>Community housing with open access</td><td>22</td><td>6</td></tr>
<tr><td>Q</td><td rowspan="3"><3%</td><td rowspan="3">Roof cover >85% corrugated iron sheets</td><td>New areas of low quality housing (built-up area <37%)</td><td>22</td><td>6</td></tr>
<tr><td>R</td><td>High density planned low quality housing (built-up area <37% AND public space >20%)</td><td>22</td><td>6</td></tr>
<tr><td>S</td><td>High density unplanned low quality housing (slums) (built-up area <37% AND public space <20%)</td><td>13</td><td>7</td><td>13</td><td>4</td></tr>
</table>

[#]**Ledant M.,** 2011 - *Socio-Economical and Infrastructural Mapping of Nairobi: Technical Report.* Nairobi: UN-Habitat.

Administrative sublocations were mapped onto each wealth group, and areas of similar neighbourhood types were identified, with the aim of maximising the geographical spread

across the city. This process identified 70 possible sub-locations, for which dominant wealth groups were calculated by extracting the proportion of population belonging to each neighbourhood class within the sub-location boundaries. The number of sub-locations to visit in each wealth group was weighted by population and the number of neighbourhood classes that went into the formation of each wealth group. A total of 33 sublocations were selected as follows: a) selection of slum and peripheral rural areas with high livestock densities, in which project activities were already being carried out (8 sub-locations); b) selection of one sub-location to represent each remaining neighbourhood class (15 sub-locations) with the highest population proportion for that class; c) selection of 10 further sub-locations to make up the target number for each wealth group, attempting to maximise both geographical spread, distribution between neighbourhood classes, and proportion of population belonging to the dominant class. For each sublocation, geographical points were selected at random within the dominant housing type, and sub-locations were fully randomised to determine the visiting order.

### Selection of households

Local administrative officials assisted in the recruitment of a household closest to each geographical point, to obtain two livestock keeping and one non-livestock keeping household per sublocation (a total of 99 households, 66 of which kept livestock). Households had to meet strict inclusion criteria of keeping either large ruminants (cattle), large monogastrics (pigs), small ruminants (goats/sheep), small monogastrics (poultry/rabbits), or no livestock species (Table 2). To ensure an equal sample of both cattle and pig-keeping households, the combination of livestock keeping households represented in each sublocation was randomised, and had to consist of either large ruminant and small monogastric, or large monogastric and small ruminant species. For sublocations in which households keeping large ruminant or large monogastric species were absent, a replacement household keeping either small monogastic or small ruminant species was recruited. A large fraction of isolates in each sublocation were obtained from a household with livestock (minimum 75%).

Table 2. Inclusion criteria for household livestock composition

| | Large ruminant | Large monogastric | Small ruminant | Small monogastric |
|---|---|---|---|---|
| Necessary and sufficient | Cattle | Pigs | Goats/sheep | Poultry or rabbits |
| Optional | Any other species | Any other species | Pigs, poultry or rabbits | Cattle, sheep or goats |
| Exclusion | None | None | Cattle | Pigs |

**Data Collection**

**Sampling of human:** In each household, the household head/owner (or a nominated member) completed a questionnaire, detailing livestock ownership (e.g. abundance of livestock species), management practices (e.g. manure disposal practices), household composition (e.g. number of occupants), and socio-economic variables. Thereafter, following an informed consent, every human member of the household was invited to contribute a faecal sample and answer questionnaires on: their age, gender and occupation, food consumption and medical history. Faecal samples were collected from people not present in the household during the visit, such as school-age children. The number of members per recruited household ranged from one to 19, including staff members and unrelated household residents. However, full participation by every member was only achieved in 20 of the 99 households. Composition of the household varied by wealth group, with households at the lower end of the wealth-scale having more children (median = 2, compared to median 1 child in wealth groups 1 and 4, and median 0 children in wealth groups 2 and 3).

**Sampling of livestock:** Rectal swabs were obtained from (up to 20) livestock species present in the household (ensuring that all species were represented). Up-to 12 different species of livestock (cattle, pigs, sheep, goats, rabbits, guinea pigs, chickens, ducks, geese, turkeys, guinea fowl and pigeons) were recruited and sampled over the course of the study (Table 3). The distribution of livestock between neighbourhood classes varied according to species. Chickens were the most common species encountered, kept by 83% of the 66 livestock-keeping households; these along with goats, rabbits and other poultry types were distributed relatively evenly across all neighbourhood classes. However, cattle and sheep were found almost exclusively in either the very wealthy areas, the very poor areas, or the areas on the eastern and western periphery of the city. The distribution of pigs was similar, except that they were not found in the higher wealth groups, although one pig-keeper in a dense new-build area (wealth group 5) was recruited.

**Sampling of wildlife:** Rodents, bats, birds and non-human primates were sampled. Rodents were trapped using medium-sized (23 cm x 7.5 cm x 9 cm) Sherman live traps (H. B. Sherman Traps Inc., Tallahassee, FL) or Victor lethal traps (Woodstream Corp., Lititz, PA) that were baited with dried fish, placed against walls throughout the household and livestock keeping facilities, and left in place for three nights. Traps were set in each household for all trapping nights and checked daily. Mist nets were set at dawn to trap birds, with nets being positioned outside the house and around livestock keeping facilities. For household compounds in which bat activity was deemed likely (as judged based on the presence of fruiting trees and/or 'flyways'), mist nets were set at dusk and monitored for two hours. Where household members reported frequent sightings of non-human primates, wire-mesh live-capture traps were pre-baited with bananas for a minimum of three days. Traps were then set, and monitored regularly for a maximum of three days. Due to large variation in the size of household compounds, trapping effort (i.e. number of traps/mist nets placed per trapping session) was maintained such that it was proportional to the size of the household compound.

Human and animal faecal samples were collected and transported on ice to one of two laboratories (University of Nairobi or Kenya Medical Research Institute) within five hours of collection. Questionnaires and data associated with samples was recorded using Open Data

Kit (ODK) Collect software, on electronic tablets, and uploaded to databases held on servers at the International Livestock Research Institute (ILRI).

Table 3: Host species distribution of samples collected

| **Host** | **Count** | **%** |
|---|---|---|
| Human | 311 | 23.2 |
| Wild birds | 245 | 18.3 |
| Chickens | 244 | 18.2 |
| Rodent | 130 | 9.7 |
| Goats | 109 | 8.1 |
| Cattle | 61 | 4.6 |
| Pigs | 49 | 3.7 |
| Rabbits | 38 | 2.8 |
| Ducks | 25 | 1.9 |
| Sheep | 25 | 1.9 |
| Bats | 20 | 1.5 |
| Guinea fowl | 18 | 1.3 |
| Wild bird roost | 15 | 1.1 |
| Geese | 14 | 1 |
| Pigeons | 13 | 1 |
| Turkey | 10 | 0.7 |
| Bat roost | 4 | 0.3 |
| Primates | 4 | 0.3 |
| Carnivore | 3 | 0.2 |
