# Supplementary material for: Population genomics of Escherichia coli in livestock-keeping households across a rapidly developing urban landscape
Source: Nat Microbiol. 2022 Mar 14;7(4):581–9. doi: 10.1038/s41564-022-01079-y (PMC8975746; doi:10.1038/s41564-022-01079-y)
Supplement: Supplementary file 2 — Reporting Summary. [file 41564_2022_1079_MOESM2_ESM.pdf]

nature research

Corresponding author(s): Eric Fevre, Mark Woolhouse

Last updated by author(s): Jan 26, 2021

Reporting Summary

Nature Research wishes to improve the reproducibility of the work that we publish. This form provides structure for consistency and transparency in reporting. For further information on Nature Research policies, see our Editorial Policies and the Editorial Policy Checklist.

Statistics

For all statistical analyses, confirm that the following items are present in the figure legend, table legend, main text, or Methods section.

n/a	Confirmed	
☐	☒	The exact sample size (*n*) for each experimental group/condition, given as a discrete number and unit of measurement
☐	☒	A statement on whether measurements were taken from distinct samples or whether the same sample was measured repeatedly
☐	☒	The statistical test(s) used AND whether they are one- or two-sided<br>*Only common tests should be described solely by name; describe more complex techniques in the Methods section.*
☐	☒	A description of all covariates tested
☐	☒	A description of any assumptions or corrections, such as tests of normality and adjustment for multiple comparisons
☐	☒	A full description of the statistical parameters including central tendency (e.g. means) or other basic estimates (e.g. regression coefficient) AND variation (e.g. standard deviation) or associated estimates of uncertainty (e.g. confidence intervals)
☐	☒	For null hypothesis testing, the test statistic (e.g. *F*, *t*, *r*) with confidence intervals, effect sizes, degrees of freedom and *P* value noted<br>*Give P values as exact values whenever suitable.*
☒	☐	For Bayesian analysis, information on the choice of priors and Markov chain Monte Carlo settings
☒	☐	For hierarchical and complex designs, identification of the appropriate level for tests and full reporting of outcomes
☒	☐	Estimates of effect sizes (e.g. Cohen's *d*, Pearson's *r*), indicating how they were calculated

Our web collection on statistics for biologists contains articles on many of the points above.

Software and code

Policy information about availability of computer code

Data collection	Field data were collected using the open source software Open Data Kit (ODK) Collect (version 1.4.1).
Data analysis	Genome analysis was performed with the following software, which is also detailed in the Methods and Supplementary Methods.<br>BBDuk (v38.46). Sequenced reads were filtered for quality and trimmed for adaptors with BBDuk (v38.46) k=19 mink=11 hdist=1 ktrim=r minoverlap=12 qtrim=rl trimq=15. Genomes were assembled using Spades v3.13.0 with the `--careful` option. Clermont phylotype of the isolates was determined using the ClermonTyping tool v1.4.1 (downloaded 20 Nov 2019). The pangenome was estimated using Roary v3.12.0 with the following options: -s -i 95 -g 100000. Acquired antibiotic resistance genes were identified from the assemblies using starAMR (v0.4.0) (https://github.com/phac-nml/staramr), with a cutoff of 95% sequence identity and a minimum of 60% alignment to the query sequence, against the ResFinder database downloaded 25 September 2019.<br><br>A core genome alignment was generated using Snippy v4.6.0 (with default settings) using EC958 as a reference genome (GCA_000285655.3). A phylogenetic analysis of the core genome alignment was performed using IQTREE (v1.6.12) -m TVM+G4 -bb 1000 -safe. The tree and metadata were visualised in iToLv4.3 (itol.embl.de). Pairwise distances were calculated using Disty McMatrixface v0.1.0 (https://github.com/c2-d2/disty) with -n 0.002.<br><br>Ad hoc core genome multi Locus sequence typing (cgMLST) was performed on genome assemblies using chewBBACA (v. 2.0.11) with the 2513 gene cgMLST profile from Enterobase (Downloaded October 2018).<br><br>A genetic distance matrix was calculated from all pairwise allelic profile comparisons using the library "ape" in R (Paradis et al., 2004). The R package "cutpointR" was used to validate this cutoff as the optimal value to differentiate pairs that occur within and between households. Custom R scripts to perform sharing distribution analysis is provided at https://git.ecdf.ed.ac.uk/epigroup/urbanzoo

For manuscripts utilizing custom algorithms or software that are central to the research but not yet described in published literature, software must be made available to editors and reviewers. We strongly encourage code deposition in a community repository (e.g. GitHub). See the Nature Research guidelines for submitting code & software for further information.

Data

Policy information about availability of data

All manuscripts must include a data availability statement. This statement should provide the following information, where applicable:

- Accession codes, unique identifiers, or web links for publicly available datasets
- A list of figures that have associated raw data
- A description of any restrictions on data availability

Whole genome sequences used in this study are available under the BioProjects with accession number PRJEB32607 and PRJEB41827. The reference genome used for mapping is E. coli strain EC958 (GCA_000285655.3). The ResFinder AMR gene database used was downloaded on 25 September 2019 from https://bitbucket.org/genomicepidemiology/resfinder_db.

Field-specific reporting

Please select the one below that is the best fit for your research. If you are not sure, read the appropriate sections before making your selection.

☐ Life sciences ☐ Behavioural & social sciences ☒ Ecological, evolutionary & environmental sciences

For a reference copy of the document with all sections, see nature.com/documents/nr-reporting-summary-flat.pdf

Ecological, evolutionary & environmental sciences study design

All studies must disclose on these points even when the disclosure is negative.

Study description	This work presented in this paper formed part of the UrbanZoo project (http://www.zoonotic-diseases.org/project/urban-zoo-project/) a Medical Research Council-funded project that aimed to utilise a landscape genetics approach to understand the movement and sharing of pathogens in a major developing city. A significant component of the UrbanZoo project was the '99 household project' which focused on sampling of households across socio-economic strata of Nairobi to investigate the role of informal livestock keeping practices as a route of zoonotic disease emergence in humans. The project was designed as a cross-sectional study, utilizing multi-stage cluster sampling by stratifying the city into 33 sublocations that were proportionately chosen to represent a gradient of socioeconomic housing types, and thus urbanization across the city. These sublocations represent the first-level of clustering. Within each sublocation, three randomly selected households represented the second level of clustering (which number 99 in total), within which samples of humans, livestock and peri-domestic wildlife represent the cluster sampling. Households were selected with the aim of maximizing the spatial distribution and diversity of livestock keeping practices across Nairobi, and were chosen to capture three main criteria: socio-economic diversity, population distribution and livestock keeping practices. Geospatial mapping data, generated as part of a technical report produced by Institut Français de Recherche en Afrique (IFRA), was used to identify 17 classes of residential neighborhood in Nairobi based on physical landscape attributes, which were subsequently verified by 817 household questionnaires. Each of the 17 classes of neighborhood were then ranked by average income and condensed into seven wealth groups. Administrative sublocations were mapped onto each wealth group, identifying a total of 70 possible sublocations, for which dominant wealth groups were calculated by extracting the proportion of population belonging to each neighborhood class within the sublocation boundaries. A total of 33 sublocations were selected to be included in the study, with the number of sublocations belonging to each wealth group chosen proportionately to the population density and the variety of neighborhood classes in each of the seven wealth groups. Final selection of individual sublocations was aimed at maximizing areas with high livestock densities, whilst ensuring coverage of other neighborhood classes and geographical spread. For each sublocation, three geographical points were selected at random within the dominant housing type, comprising of: two livestock keeping and one non-livestock keeping household. A total of 99 households, 66 of which kept livestock were visited. Livestock keeping households had to meet strict inclusion criteria of: (i) keeping small livestock only (small ruminants - goats/sheep, small monogastrics - poultry/rabbits), and (ii) large livestock (large ruminants (cattle), large monogastrics (pigs), with or without small livestock. To ensure an equal sample of both cattle and pig-keeping households, the combination of livestock keeping households represented in each sublocation was randomised, and had to consist of either large ruminant and small monogastric, or large monogastric and small ruminant species. For sublocations in which households keeping large ruminant or large monogastric species were absent, a replacement household keeping either small monogastic or small ruminant species was recruited. The order in which sublocations were visited was randomized. Within the sublocations, local administrative leaders assisted in recruitment, which was carried out a few days before the sampling date. The three pre-selected geographical points were identified on the ground, and the nearest three households that met the inclusion criteria identified.
Research sample	A total of 1,338 samples were collected as part of this study including: 311 samples from humans, 421 samples from 63 wildlife species [comprising of wild birds (n=245), rodents and bats(n=130)]. 606 samples from 13 species of livestock that can be grouped into poultry (n=324), goat and sheep (n=109),cattle (n=61), 94 pig (n=49) and rabbit (n=38)isolates. The isolates were distributed across 33 geographic sublocations spanning the entire urban area of Nairobi. Humans were sampled irrespective of age and gender. Food producing animals (including cattle,goats, poultry, pigs and rabbits) were sampled as they represent a direct link to humans either through food, direct contact or shared habitats. Avian (wild birds and bats), rodents and non-human primates were the selected wildlife hosts in this urban study system, since they are diversely and widely distributed across urban landscapes, demonstrating epidemiological and ecological responses to land-use change, and interacting closely with livestock and humans
Sampling strategy	Owing to the design of this study – genetic analysis of E .coli population in a unstudied urban population for which it is challenging to predict significance in advance, we were unable to generate robust statistical power calculations or sample sizes posed in the study. As such, the number of samples (human, livestock and wildlife) varied according to the household sizes. Due to large variation in the size of household compounds, trapping effort of wildlife species (i.e. number of rodent traps placed per trapping session) was maintained such that it was proportional to the size of the household compound, and thus standardized across households.

Data collection	Sampling of human: In each household, the household head/owner (or a nominated member) completed a questionnaire, detailing livestock ownership (e.g. abundance of livestock species), management practices (e.g. manure disposal practices), household composition (e.g. number of occupants), and socio-economic variables. Thereafter, following an informed consent, every human member of the household was invited to contribute a faecal sample and answer questionnaires on: their age, gender and occupation, food consumption and medical history. Faecal samples were collected from people not present in the household during the visit, such as school-age children. The number of members per recruited household ranged from one to 19, including staff members and unrelated household residents. However, full participation by every member was only achieved in 20 of the 99 households. Composition of the household varied by wealth group, with households at the lower end of the wealth-scale having more children (median = 2, compared to median 1 child in wealth groups 1 and 4, and median 0 children in wealth groups 2 and 3).<br><br>Sampling of livestock: Rectal swabs were obtained from (up to 20) livestock species present in the household (ensuring that all species were represented). Up-to 12 different species of livestock(cattle, pigs, sheep, goats, rabbits, guinea pigs, chickens, ducks, geese, turkeys, guinea fowl and pigeons) were recruited and sampled over the course of the study(Table xx). The distribution of livestock between neighbourhood classes varied according to species.Chickens were the most common species encountered, kept by 83% of the 66 livestock-keeping households; these along with goats, rabbits and other poultry types were distributed relatively evenly across all neighbourhood classes. However, cattle and sheep were found almost exclusively in either the very wealthy areas, the very poor areas, or the areas on the eastern and western periphery of the city. The distribution of pigs was similar, except that they were not found in the higher wealth groups, although one pig-keeper in a dense new-build area (wealth group 5) was recruited.<br><br>Sampling of wildlife: Rodents, bats, birds and non-human primates were sampled. Rodents were trapped using medium-sized (23 cm x 7.5 cm x 9 cm) Sherman live traps (H. B. Sherman Traps Inc., Tallahassee, FL) or Victor lethal traps (Woodstream Corp., Lititz,PA) that were baited with dried fish, placed against walls throughout the household and livestock keeping facilities, and left in place for three nights. Traps were set in each household for all trapping nights and checked daily. Mist nets were set at dawn to trap birds, with nets being positioned outside the house and around livestock keeping facilities. For household compounds in which bat activity was deemed likely (as judged based on the presence of fruiting trees and/or 'flyways'), mist nets were set at dusk and monitored for two hours. Where household members reported frequent sightings of non-human primates, wire-mesh live-capture traps were pre-baited with bananas for a minimum of three days. Traps were then set, and monitored regularly for a maximum of three days. Due to large variation in the size of household compounds, trapping effort (i.e. number of traps/mist nets placed per trapping session) was maintained such that it was proportional to the size of the household compound.<br>Human and animal faecal samples were collected and transported on ice to one of two laboratories (University of Nairobi or Kenya Medical Research Institute) within five hours of collection. Questionnaires and data associated with samples was recorded using Open Data Kit (ODK) Collect software, on electronic tablets, and uploaded to databases held on servers at the International Livestock Research Institute (ILRI). Field teams involved in data collection consisted of two clinical officers, and one to three veterinarians/ animal health workers - all Kenyan nationals, fluent in both Kiswahili and English, and participants could opt to complete the questionnaires in either of these languages.
Timing and spatial scale	Field data was collected between September 2015 and September 2016 across the city of Nairobi. Triplets of households with each sublocation were sampled within the same week. All field data collection for each household was conducted on the same day.
Data exclusions	Isolates deemed not to be E. coli, on the basis of biochemical testing or whole genome sequencing, were removed from the dataset.
Reproducibility	Standard epidemiological, laboratory (microbiology and sequencing) and analytical approaches were used throughout the study and all data used in this study is available in open-source platforms. Sampling effort was maintained such that it was proportional to the household composition and size and sampling was standardised (being conducted by the same team of veterinarians and clinicians). Field samples were sent to one of two laboratories (University of Nairobi and Kenya Medical Research Institute) for microbial culture, and all efforts were undertaken to ensure that this did not introduce bias into the study. Protocols were standardized between laboratories. All analytical processed were conducted in R Statistical environment and code is provided.
Randomization	Participants were not allocated into experimental groups. However, geographical points used to select households within each sublocation were distributed at random. The combination of livestock keeping households represented in each sublocation was randomized, and had to consist of either large ruminant and small monogastric, or large monogastric and small ruminant species. The order in which sublocations were visited for data collection between September 2015 and September 2016 was randomized. One purified E. coli isolate per original sample grown was selected at random. During analysis isolates were grouped according to the host animal species (e.g. human/poultry), household source (e.g. same household).
Blinding	Since the "groups" in question were host species (humans, livestock and wildlife and household it was not feasible to blind samplers to either. No attempts were made at blinding during microbiological processing or DNA extraction. Library preparation and whole genome sequencing was performed in a separate country and personnel, who were blind to the groups. No blinding was attempted during data analysis.

Did the study involve field work? ☒ Yes ☐ No

Field work, collection and transport

Field conditions	Climatic and weather conditions were not investigated in this study however to account their possible impact on study outcomes, field work was conducted over the course of one year, and as such precipitation and temperature varied over the course of the study. Topographical and natural habitat conditions differ markedly across the city – e.g greener and lush in the South and West, and savannah biome in the East and North – and as such between our study households. Fieldwork was conducted between 7 am and 10 am with the exception of bat sampling that happened between 6:00 pm and 7:30 pm.
Location	Sampling was conducted in households across the city of Nairobi, Kenya. Nairobi lies just below the equator with a latitude of -1.286389, and longitude is 36.817223 and lies at 1,795 metres (5,889 ft) above sea level.

Access & import/export	The collection of data adhered to the legal requirements of the International Livestock Research Institute (ILRI), and Government of Kenya regulations. Permission to access study locations was obtained from the administrative authorities and the National Commission for Science, Technology and Innovation in Kenya. Additional permits to sample livestock and wildlife were obtained from the Directorate of Veterinary Services, the National Museums of Kenya and Kenya Wildlife Service respectively. E. coli DNA was exported from Kenya to The Wellcome Trust Centre for Human Genetics, Oxford, UK under a Kenyan Ministry of Agriculture license RES/POL/VOL XXIV/72, in adherence to Nagoya protocol requirements.
Disturbance	Sampling was non destructive or harmful to participants or environments involved.

Reporting for specific materials, systems and methods

We require information from authors about some types of materials, experimental systems and methods used in many studies. Here, indicate whether each material, system or method listed is relevant to your study. If you are not sure if a list item applies to your research, read the appropriate section before selecting a response.

Materials & experimental systems

n/a	Involved in the study
☒	☐ Antibodies
☒	☐ Eukaryotic cell lines
☒	☐ Palaeontology and archaeology
☐	☒ Animals and other organisms
☐	☒ Human research participants
☒	☐ Clinical data
☒	☐ Dual use research of concern

Methods

n/a	Involved in the study
☒	☐ ChIP-seq
☒	☐ Flow cytometry
☒	☐ MRI-based neuroimaging

Animals and other organisms

Policy information about studies involving animals; ARRIVE guidelines recommended for reporting animal research

Laboratory animals	Study did not involve laboratory animals
Wild animals	Once caught, all birds, and all but two bats caught per trapping session, were live-sampled in the field under manual restraint, before being released unharmed. All live rodents (except for individuals belonging to the genus Cricetomys, which were live-sampled under anaesthesia) and up to two bats caught per trapping session were transferred back to a biosafety level three (BSL3) laboratory at ILRI. Trapped rodents and bats were placed in containers that were resistant to escape, provided adequate ventilation and protection from the elements. Holding containers were transported via project vehicles that were decontaminated after use. at the labortaory, he animals were humanely euthanised by cardiac puncture under isoflurane anaesthesia and a full post-mortem examination then performed, with fresh faeces being collected from the rectum. Rodents caught in lethal traps were also necropsied in the laboratory following the same protocols. Faecal samples were collected non-invasively from small carnivores, by keeping them in the trap for a maximum period of twelve hours. Non human primates were anaesthetised where trapped, using a combination of Medetomidine and Ketamine (under the supervision of a Kenya Wildlife Service veterinary officer), and morphometric data and a suite of biological samples (including faeces if available, or a rectal swab) were collected from each animal. The primate was carefully monitored throughout, and anaesthesia reversed using Atipamezol. Carnivores and NHPs were released unharmed at an appropriate time of day, from the same location at which they were trapped. Rodents were euthanized humanely for two reasons, (i) because they were trapped within people's households and release of species that are deemed as pests (and a potential public health hazard) would not have been a viable option, (ii)In order to collect a fresh fecal samples via post-mortem.
Field-collected samples	Human and animal faecal samples were transported from the field on ice (4 degrees) to the laboratory within 5 h of collection. Extracted DNA was stored in -20 degree freezers and transported on dry ice to Oxford University for whole genome sequencing.
Ethics oversight	The collection of data adhered to the legal requirements of the Government of Kenya. The International Livestock Research Institute Institutional Research Ethics Committee is registered and accredited by the National Commission for Science, Technology and Innovation in Kenya. Livestock samples were obtained under the approval of the ILRI Institutional Animal Care and Use Committee (Reference ILR-IACUC2015/18) and permits obtained from the Directorate of Veterinary Services. Wildlife were trapped under approval of an ILRI Institutional Animal Care and Use Protocol (IACUC2015/12), and permits were obtained from the National Museums of Kenya and Kenya Wildlife Service

Note that full information on the approval of the study protocol must also be provided in the manuscript.

Human research participants

Policy information about studies involving human research participants

Population characteristics	In each of the 99 households across Nairobi that participated in the study, the human participants that contributed faecal samples had their age, gender, occupation, food consumption and medical history recorded on a questionnaire. The number of members per recruited household ranged from one to 19, including staff members and unrelated household residents. However, full participation by every member was only achieved in 20 of the 99 households. Composition of the household varied by wealth group, with households at the lower end of the wealth-scale having more children (median = 2,

compared to median 1 child in wealth groups 1 and 4, and median 0 children in wealth groups 2 and 3). Human specific covariates such as age, gender were not included in the analysis.

Recruitment	Within a study sub-location three randomly three points (each representing a household type - two livestock keeping and one non livestock keeping) in GIS were dropped. The nearest household to that point (within the dominant household type) was located - in most cases non-livestock keeping. Local administrative officials assisted to locate the nearest (Euclidian distance) households to that first selected that represent the two other classes, in most cases livestock keeping households. In each household, the household head/owner (or a nominated member) completed a questionnaire, detailing livestock ownership (e.g. abundance of livestock species), management practices (e.g. manure disposal practices), household composition (e.g. number of occupants), and socio-economic variables. Thereafter, following an informed consent, every human member of the household was invited to contribute a faecal sample and answer questionnaires on: their age, gender and occupation, food consumption and medical history. Faecal samples were collected from people not present in the household during the visit, such as school-age children. No bias were identified that could impact on the results.
Ethics oversight	The International Livestock Research Institute Institutional Research Ethics Committee is registered and accredited by the National Commission for Science, Technology and Innovation in Kenya, and approved by the Federal wide Assurance for the Protection of Human Subjects in the USA. Ethical approval for human sampling and data collection was obtained from the ILRI Institutional Research Ethics Committee (ILRI-IACUC2015/09).

Note that full information on the approval of the study protocol must also be provided in the manuscript.
